# Editorial: Vibration-based robot locomotion

**DOI:** 10.3389/frobt.2024.1508130

**Published:** 2024-10-29

**Authors:** Murat Reis, Silvio Cocuzza, Alberto Doria, Nurettin Gökhan Adar

**Affiliations:** ^1^ Department of Mechanical Engineering, Bursa Uludağ University, Bursa, Türkiye; ^2^ Department of Industrial Engineering, University of Padua, Padua, Italy; ^3^ Department of Mechatronic Engineering, Bursa Technical University, Bursa, Türkiye

**Keywords:** vibration, locomotion, robot, soft, elastic, resonance

## Introduction

The locomotion of robots or living beings exhibits periodic characteristics, similarly to the vibrational motion of elastic bodies. The constant-speed locomotion of living beings, such as human walking, horse running, kangaroo jumping, swimming, etc., or robots walking on a flat trajectory has a nearly perfect periodic characteristic. Each limb repeats the same periodic motion at the frequency of the locomotion. If so, this can be explained by the periodic shape changes during the locomotion of complex multi-limbed bodies, activating the natural vibration behavior of rigid bodies with elastic connections or of elastic bodies, resulting in an under-actuated locomotion. Moreover, legged locomotion methods such as walking, running, and jumping are energy inefficient due to impact loads. This problem can be overcome using elastic leg structures and elastic actuators in the joints. However, using elastic structural elements requires an actuation method compatible with the natural vibrational frequencies of these elements. These elastic mechanisms can create surprisingly energy-efficient and stable locomotion mechanisms. This Research Topic mainly aimed to compile recent studies emphasizing vibration as a control and actuation method of mobile robots. There were valuable submissions to this Research Topic, and as a result of the referee evaluations, the following four articles were deemed worthy of publication. We want to thank the authors and referees who contributed to the study.

1. *Bidirectional Locomotion of Soft Inchworm Crawler Using Dynamic Gaits* (Du et al.)

This paper investigates a crawling mechanism that enables a soft robot to perform bidirectional locomotion using body deformation. The authors propose two different differential friction forces integrated into the robot’s body structure, allowing the robot to generate two different motion directions as the body deforms. With the proposed dynamic gaits, the robot can move in multiple directions with a simple system configuration and a minimalist actuation input. This paper provides an interesting example of how soft structure vibrations can be used for challenging robotic tasks.

2. *Controlling the motion of gas-lubricated adhesive disks using multiple vibration sources* (Jia et al.)

This paper discusses a new approach for robots that can generate adhesive force on a surface and move freely by overcoming their gravity. The authors developed a system that allows adhesion while moving along a surface using multiple vibration sources. The experiments show that gas-lubricated adhesive disks can move on the surface without any auxiliary operating system. This study constitutes a step towards constructing small-sized wireless robots that can overcome gravity and move freely in a general environment.

3. *Locomotion characteristics of a wheeled vibration-driven robot with an enhanced pantograph-type suspension* (Korendiy et al.)

This study considers a new design in which a wheeled robot is equipped with an enhanced pantograph-type suspension. The research includes mathematical modeling, computer simulation, and experimental tests of the robot’s locomotion conditions. The results show the time dependencies of different kinematic parameters in the robot operating conditions and reveal the potential of this robot design for use in applications such as inspection and cleaning of pipelines. The study provides an essential roadmap by suggesting further analysis of power consumption, average speed, and operating efficiency.

4. *Robust self-propulsion in sand using simply controlled vibrating cubes* (Liu et al.)

This paper explores a self-propulsion mechanism via vibration as an alternative solution to the difficulty of moving on loose granular surfaces. The authors developed a cube-shaped robot with an embedded vibration motor and conducted various experiments on granular surfaces. The results show that such a robot can move faster and more stably on granular surfaces. The numerical simulations confirm that the robotmovement is driven by oscillations triggered at the distance from the center of mass. This simple design and control structure highlights vibratory locomotion as a valuable way to provide reliable movement on granular surfaces.

## General assessment

These four studies present various innovative vibration-based solutions to enhance the locomotion capabilities of different robotic systems. The proposed innovative designs, such as soft robots, gas-lubricated adhesive disks, improved suspension systems, and vibrating cubes, aim to increase the efficiency of robots regardless of the type of soil. Each study makes significant contributions to the field of robotics by combining applied and theoretical knowledge to enhance the mobility of mobile robots in complex environmental conditions. In the future, the findings of these studies may inspire more advanced and efficient robot designs and applications.

